# “What impact does having a diagnosis of an inherited cardiac condition have on children and young people’s physical activity and quality of life?” A scoping review

**DOI:** 10.1007/s00431-025-06658-9

**Published:** 2026-01-06

**Authors:** Scott Kendall, Veda Maha Kyla Murugaperumal, Andrea Greco, Terence Prendiville, Martin Dempster, Pascal McKeown, Frank Casey

**Affiliations:** 1 Wellcome Wolfson Institute for Experimental Medicine, Queen’s University Belfast, Belfast, Northern Ireland UK; 2 Paediatric Cardiology Department, Royal Belfast Hospital for Sick Children, Belfast, Northern Ireland UK; 3 School of Medicine, Dentistry and Biomedical Sciences, Queen’s University Belfast, Belfast, Northern Ireland UK; 4 Paediatrics Department, University of Barcelona, Barcelona, Spain; 5 Inherited Cardiac Diseases and Sudden Death Unit, Hospital Sant Joan de Deu Barcelona, Barcelona, Spain; 6 Paediatric Cardiology Department, Children’s Health Ireland at Crumlin, Dublin, Ireland; 7 School of Psychology, Queen’s University Belfast, Belfast, Northern Ireland UK; 8 School of Medicine, University of Ulster, Belfast, Northern Ireland UK

**Keywords:** Heart, Channelopathies, Cardiomyopathies, Physical activity, Quality of life

## Abstract

Inherited cardiac conditions (ICCs) are an umbrella group of genetic disorders affecting the heart. They are life-long conditions that are often diagnosed through family screening or after cardiac events. For many years, it has been customary practice to restrict physical activity (PA) in children to prevent ventricular arrhythmia or sudden cardiac death. This scoping review aims to identify and analyse the current literature on the impact that having a diagnosis of an ICC has on a young person’s PA and quality of life (QoL). This scoping review follows the methods of Arksey and O’Malley. Studies concerned with QoL and PA published between 1957 and 2025 were included. Literature searches were conducted across the following databases: Medline (Ovid), Scopus, Web of Science, Embase (Ovid) CINAHL, and PsycINFO. The data was extracted and charted focussing on aims, study populations, measures, and results. Data was then summarised with a descriptive summary as well as visual mapping and descriptive statistics. The review was conducted using the Preferred Reporting Items for Scoping Reviews guidelines. From a total of 1358 identified articles, 27 articles were included in the final review. There were twenty quantitative articles, three mixed method, and four qualitative studies included. Reduced QoL and mental health issues were common in this cohort. Children with ICCs had lower reported and measured PA in comparison to healthy peers. Lower PA was associated with reduced QOL amongst patients with ICCs. Themes identified in qualitative studies included activity restriction, fear of cardiac events, a need for improved communication by healthcare professionals, re-orientation, and coping. *Conclusion*: Children and young people with ICCs appear to have poorer QoL and engage in less PA than healthy peers. Empowering them to safely engage in PA should be a priority for healthcare providers. Consistent outcome measure across future studies would improve wider applicability of the findings.

**Table:** 

**What is Known:** •* Children and young people with inherited cardiac conditions are subject to physical activity restrictions*. •* Being affected by an inherited cardiac condition is associated with poorer heatlth related quality of life*.
**What is New:** •* Children and young people have lower reported and measured PA in comparison to healthy peers (in excess of that advised by restrictions)*. •* Lower Physical Activity is associted with reduced Quality of life amongst young people with ICCS*.

**Supplementary information:**

The online version contains supplementary material available at 10.1007/s00431-025-06658-9.

## Introduction

Inherited cardiac conditions (ICCs) encompass a broad spectrum of genetic disorders that impact cardiac function. These include both channelopathies and cardiomyopathies. Channelopathies involve dysfunction of the ion channels responsible for generating and propagating cardiac action potentials, increasing susceptibility to serious arrhythmias. The most common example is congenital long QT syndrome (LQTS) [1]. Cardiomyopathies, on the other hand, affect cardiac myocyte structure and contractile function and can lead to heart failure, obstruction of blood flow, arrhythmias, and, in severe cases, necessitate heart transplantation. Common types include hypertrophic cardiomyopathy (HCM) and dilated cardiomyopathy (DCM) [2]. Other examples of ICCs include aortopathies [3] and familial hypercholesterolaemia [4]; however, these conditions have not been included in this scoping review, as with some exceptions (e.g. weightlifting/severe aortic dilatation in Marfan’s syndrome), they are not generally associated with stringent physical activity (PA) restrictions [5].

Historically, these conditions were considered absolute or relative contraindications to participation in sports, due to concerns over sudden cardiac death (SCD) or serious arrhythmic events, collectively termed breakthrough cardiac events (BCEs) [6, 7]. More recently, emerging data have started to question the blanket assumption that PA is inherently dangerous for individuals with ICCs [8–10], leading to a gradual trend toward more permissive guidelines [2, 5, 11]. This trend is timely, as rates of childhood obesity continue to rise, in part due to physical inactivity and increased caloric intake [12]. There is also evidence that engaging in regular PA is linked to improved quality of life (QoL) in young people [13]. Regardless of views on competitive sports, healthcare professionals should recognise the broader health benefits of regular physical activity. It is also important to distinguish between moderate exercise, as recommended by public health bodies, and intensive training aimed at high-level athletic performance [5, 11].

Intriguingly some studies have also suggested that exercise may be protective for individuals with HCM and improve functional status [14, 15]. It is also true that obesity has been shown to worsen HCM disease progression [16]. Therefore, it is conceivable that by trying to protect patients from risk, reducing their activities has had the opposite effect. It is important to note that, while cardiomyopathy variant status is common (e.g. 1 in 500 for HCM), severe phenotypic expression of cardiomyopathies in children, which can be life-threatening, is uncommon, with an estimated incidence of approximately 1 in 100,000 person-years [17]. In contrast, LQTS is more prevalent, affecting an estimated 1 in 2000 to 1 in 2500 individuals [18]. A significantly larger number of children will be identified as having a pathogenic variant in a disease-associated gene without exhibiting clinical symptoms—a state referred to as genotype-positive, phenotype-negative [19]. Accurately assessing risk in these individuals often requires complex diagnostic evaluation and is informed by a rapidly evolving body of evidence [2]. In some cases, children, and families—particularly those recently affected by a sudden cardiac event or bereavement—may not fully comprehend the clinical implications of their diagnosis. As a result, they may adopt unnecessarily restrictive PA behaviours, despite the absence of overt disease expression.

This scoping review aims to explore the existing body of research on PA and QoL in children and adolescents with ICCs. By mapping the literature in this area, we intend to highlight prevailing themes, clarify current knowledge, and identify key research gaps. We hope this review will serve as a foundation for future studies focused on PA and its impact on QoL in this population.

## Methods

A scoping review was conducted following the methods of Arksey and O’Malley (1) and further evolved by Levac (2) and Colquhoun (3). It is reported according to the Preferred Reporting Items for Systematic Reviews and Meta-Analyses (PRISMA) extension for scoping reviews [4, 5].

### Defining the research question

The search was designed to answer the following question: “What impact does having a diagnosis of an inherited cardiac condition have on children and young people’s physical activity and quality of life?”.

### Protocol and registration

A scoping review protocol was registered on Open Science Framework on the 27th of March 2025 [6], and it was posted as a pre-print on Research Square on the 14th of April 2025 [7].

### Eligibility criteria

Studies were eligible for inclusion if published between 1957 and 2025, and all studies that reported on QoL, PA or both in the paediatric ICC population were included. Studies were deemed eligible if they were randomised controlled trials, pilot studies, observational cross sectional, cohort studies, and qualitative studies. Case reports were not included. Studies that were only reported in the adult population were not included. Quantitative studies that measured mental health disorder features were also included due to the profound effect mental health conditions can have on a young person’s QoL [20]. Articles not published in English or from the grey literature were excluded.

### Search terms

The following search terms were used:

Physical Activity OR Exercise OR Sports AND Quality of Life OR QoL AND Genetic Diseases, Inborn OR Inherited Cardiac Condition OR Long QT Syndrome OR Cardiomyopathy AND Child OR Young OR Adolescent OR Paediatrics OR Pediatrics (see Supplementary Material 1).

### Information sources

Searches were conducted on Medline (Ovid), Scopus, Web of Science, Embase (Ovid) CINAHL, and PsycINFO, and the final search was conducted on the 24th of March 2025.

### Search results

**Fig. 1:**
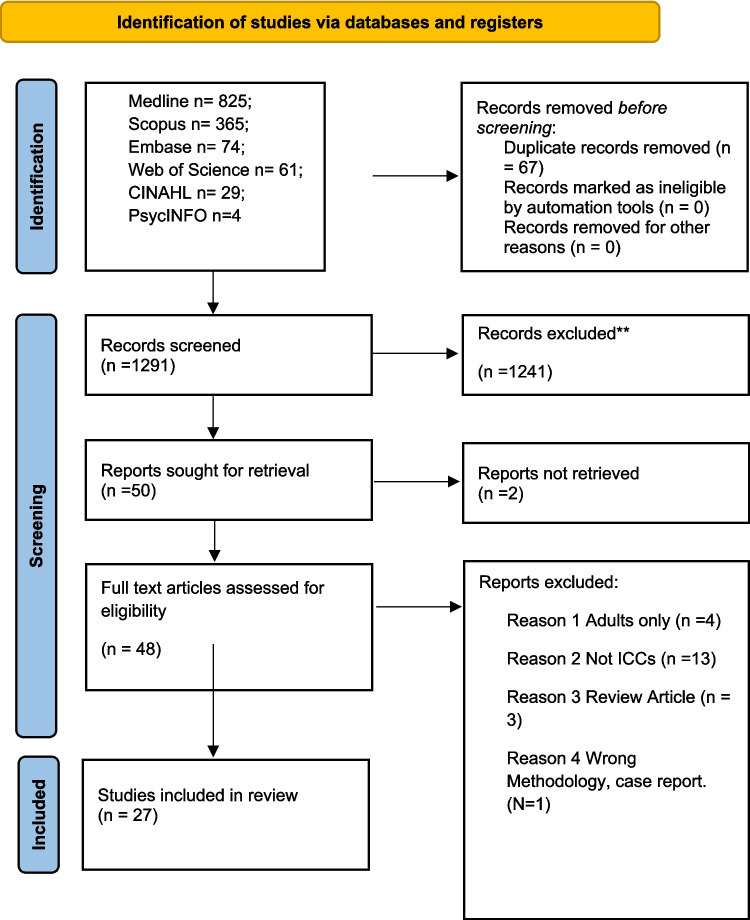
PRISMA flow diagram

The results of searches were uploaded to the review website Rayyan and then underwent title and abstract screening followed by full text screening based on the eligibility criteria by three researchers independently (SK/VKMK/AG), and conflicts were resolved by discussion. Following abstract and title screening and then full text screening, a total of 27 articles were included. A preferred reporting item for systematic reviews and meta-analyses (PRISMA) flow chart demonstrating study selection at each stage is included (see Fig. 1).

### Extraction and charting the data

A data extraction form was designed and used to extract key information from each study. The researchers met after the first ten articles to ensure consistency and then met again once the data extraction was complete. The data extraction form was iterative in nature. Two of the reviewers (SK/VKMK) independently charted the data and then met to compare forms, with discussion about any differences, in the event of any disagreements, this was resolved with third party involvement (FC). The results were then pooled to one master document which was referred to for writing the review (see Supplementary Material 2).

## Results

### Study characteristics

#### Country and year of publication

**Fig. 2:**
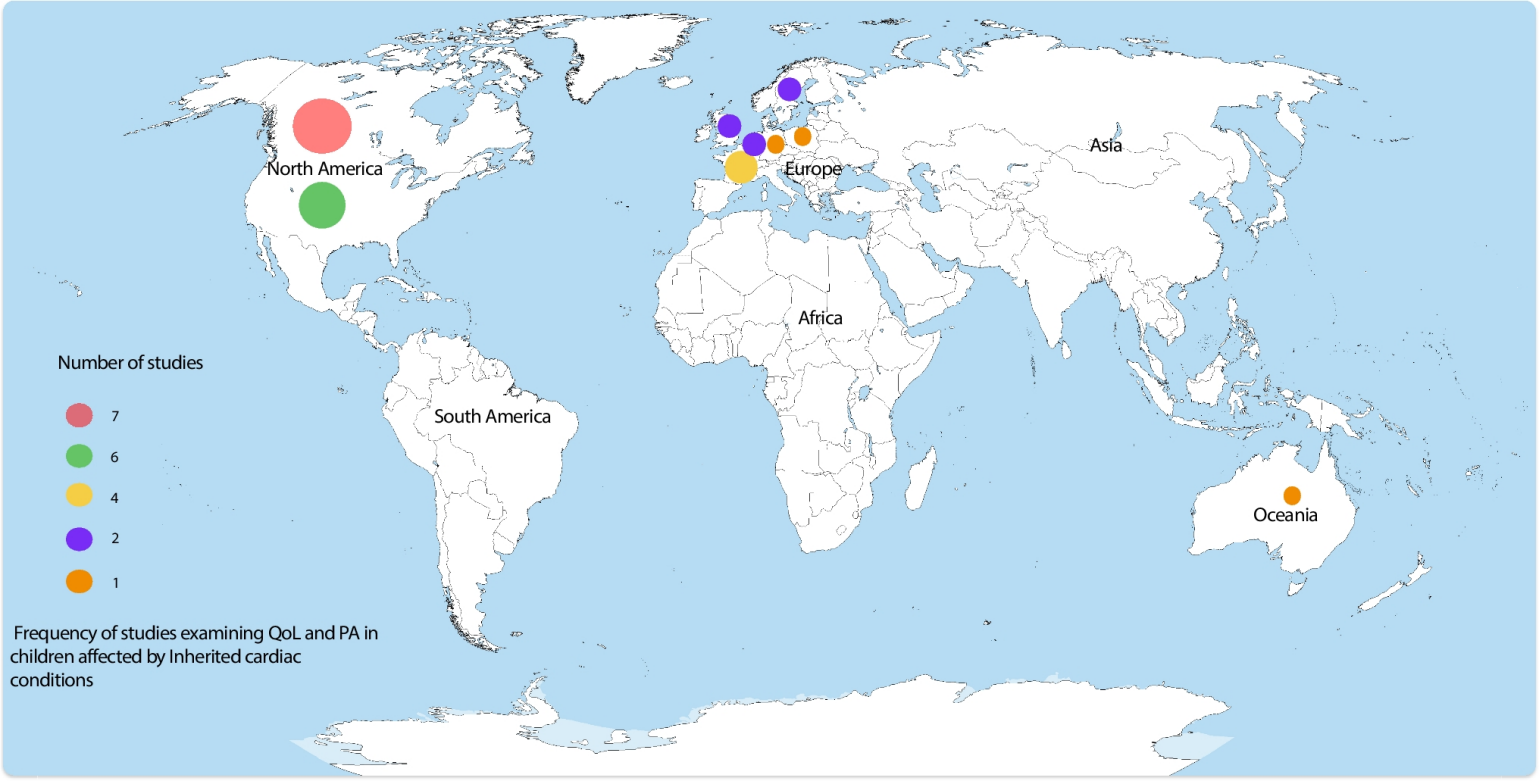
Frequency of studies examining quality of life and physical activity in children affected by inherited cardiac conditions worldwide

A total of 27 studies were included, conducted across a range of countries: Canada was the most common (*n* = 7), followed by the USA (*n* = 6), France (*n* = 4), Sweden (*n* = 2), the UK (*n* = 2), the Netherlands (*n* = 2), and one study each from Poland, Australia, and Germany; one additional study was conducted across both the UK and the USA (see Fig. 2). The earliest study included was published in 2004, with the majority (*n* = 18) being published in 2015 or later. Only 6/27 (22%) of studies reported the participant ethnicity, in all studies most participants were white (range 59%–91%).

#### Method

There were twenty quantitative articles, three mixed method, and four qualitative studies included. Sample size for quantitative and mixed methods studies ranged from 8 to 355 participants, while qualitative sample size ranged from 12 to 28 participants.

#### Age

Most studies were conducted solely in participants aged 18 or under, and one study included patients up to the age of 35. Most studies had a wide age range, e.g. from 6 to 18, and four studies recruited adolescents [12–18] solely.

#### Tools used to measure QoL

Several scales were used to measure QoL; PedsQL 4.0 [21] was used most commonly (8 studies), others included PedsQL Cardiac Module [22] (2 studies), Kidscreen 52 [23] (1 study), Paediatric Cardiac Quality of Live Inventory (PCQLI) [24] (3 studies), Child Health Questionnaire (CHQ) [25] (2 studies), Lindström QoL Questionnaire [26] (1 study). All these tools are validated for use in children; most of them also have parental modules. The PedsQL Cardiac Module and PCQLI are specifically designed for children with heart disease.

#### Tools used for measuring psychological challenges

Anxiety questionnaires were frequently employed in the included studies; these included anxiety of the study participants, disease related anxiety, and parental anxiety. Behavioural problems were also screened for in some studies. Unless otherwise stated, each scale was only used in one study. Anxiety questionnaires used included the Childhood Anxiety Sensitivity Index (CASI [27], Revised Child Anxiety and Depression Scale (RCADS) [28], Screen for anxiety related emotional disorders (SCARED) [29], Revised Children’s Manifest Anxiety Scale (RCMAS) [30], and Multidimensional Anxiety Scale for Children [31]. A disease specific anxiety questionnaire employed in one study was the Cardiac related Anxiety Questionnaire for children (CAQ-C) [32].

Questionnaires investigating behavioural problems included the Strengths and Difficulties Questionnaire [33], Child behaviour checklist [34] (two studies) Youth Self Report (YSR) [35], and Behaviour Assessment System for Children (BASC) [36].

Parental anxiety Tool used was the State-Trait Anxiety Inventory [37]. Depression questionnaires included the following: Reynolds Adolescent/Child Depression Scale (RADs) [38]; Depression and Diagnostic System for psychiatric disorders in childhood and adolescence (Translated from the original German) (DISYPS-KJ) [39].

#### Other related tools

Health status was measured of participants in one study by using the functional status tool (FSII) [40].

#### Tool used for measuring PA and physical fitness

Tools used for measuring PA in the included studies included subjective recall in the form of validated surveys including the Physical Activity Questionnaire for Children (PAQ-C) [41] (2 studies), the Ricci and Gagnon Physical Activity Questionnaire [42], the Physical Activity and Leisure Motivation Scale for Youth (PALMS-Y) [43], and Habitual Activity Estimate Scale (HAES) [44]. All these questionnaires had been validated prior to use in these studies. PA was also measured by wearing accelerometers in eight studies (wear time ranged from 7 to 14 days), and six studies used the ActiGraph (GT3x, GT9X; ActiGraph, Pensacola, FL) accelerometers which have been demonstrated to be comparable for PA measurement between different activity monitors [45]. One study used Fitbits devices (Fitbit, San Francisco, CA) to measure PA, citing improved adherence compared to Actigraph systems [46]. Another study used the Actiheart activity monitor (AH2; CamnTech, Cambridge, UK).

Physical fitness was assessed in a minority of studies—this included parameters such as weight, height, and body mass index (BMI). Exercise testing in the form of exercise stress testing (EST) [47] was performed in one study, and cardiopulmonary exercise testing (CPET) [48] was performed in four studies. Two studies also examined muscle architecture by ultrasound [48] and grip strength [49].

#### Qualitative methods

Semi structured interviews (SSIs) were conducted in the qualitative and mixed method studies included. These studies involved performing an interview, which was either recorded and/or transcribed, and the investigators then analysed the data for themes. Some studies used interviews simply to confirm adherence with exercise restriction, whilst others used it to develop models to describe the psychological impact on diagnosis of an ICC and impact on daily life.

#### Setting

All but one of the studies took place in a hospital environment (typically a paediatric cardiology or cardiogenetic clinic). The other study was conducted by telephone.

## Findings

### Quality of life

#### Generic QoL quantitative measurements

When compared to normative data in healthy children, most studies found that children affected by an ICC had lower QoL scores as measured by patient or child report [50–54]. Two studies making such a comparison found no significant difference in scores [55, 56]. Different QoL measures were used between studies making direct comparison difficult. The most used tool was the PedsQL 4.0 which is a generic QoL tool which asks 23 questions regarding physical and psychosocial (subdivided into emotional, social, and school) functioning [21]. Children affected with both LQTS [50] and cardiomyopathy [52, 54, 57] had significantly lower scores compared to normative data as measured by the PedsQL 4.0 tool. The CHQ is another QoL tool which asks 50 questions sub-divided into 14 health concepts giving a score ranging from 0 to 100 for both physical and psychosocial health [25]. One study found the CHQ was lower by parental report than normative data in children with cardiomyopathies [53]. The Lindström QoL questionnaire incorporates objective and subjective aspects of three spheres (external, interpersonal, and personal), and in a small cohort of 13 children with HCM, there was no significant difference in scores between patients and 41 healthy controls [55]. The Kidscreen-52 is a further QoL questionnaire which has ten sub-domains, including aspects such as physical well-being, psychological well-being, and the wider context such as acceptance by peers and autonomy [58]. In a mixed cohort study of patients who were positive carriers for ICCs, there was no significant difference in Kidscreen-52 scores compared with normative data; this study did include many patients with familial hypercholesterolaemia and interestingly there was a discrepancy between self-reported and parental QoL with parents tending to score lower than their children [56].

#### Disease specific QoL quantitative measures

Several QoL scores have been developed to measure and describe specific cardiac disease impact, and two such scores were used in included studies, the PedsQL Cardiac 3.0 Module and the PCQLI. The PedsQL 3.0 cardiac Module is a 28-question scale broken into six sub-domains: symptoms, perceived physical appearance, treatment anxiety, cognitive problems, communication, and treatment barriers. It can be answered by both children and parent proxies. Patients affected by cardiomyopathy have been shown to have significantly lower total scores than patients with a family history on both child [52] and parent report [54]. The PCQLI is a cardiac disease specific paediatric QoL scale which measures two subdomains: disease impact and psychosocial impact giving a total score out of 100 with higher scores denoting better perceived QoL [24]. In two studies, patients with LQTS with implanted ICDs demonstrated lower PCQLI scores compared to those without devices, and their scores were similar to those of patients with repaired Tetralogy of Fallot [50, 51].

#### Psychological challenges quantitative measures

Some studies examined psychological challenges, particularly anxiety, in this population. One study examined cardiac focused anxiety, as well as general anxiety in children attending an ICC clinic; it employed three questionnaires the CAQ-C, CASI, and RCADS. These are all self-reported questionnaires that measure cardiac related anxiety, anxiety sensitivity (individuals’ fear of anxiety related symptoms), and general anxiety score, respectively. It found that children affected had significantly more cardiac related anxiety as measured by the CAQ-C than children with a family history and unaffected controls, and this correlated with CASI scores. There was no significant difference in general anxiety between groups as measured by the RCADS [32]. A mixed cohort study was performed examining QoL (using the PedsQl 4.0), anxiety (using the multidimensional scale for children), and broader psychosocial health (using the Behavioural assessment for children). This first two scales were answered by both parents and children and, interestingly, found that there were no significant associations between parent and child reported anxiety or QoL [59]. Another study compared both maternal and child anxiety when affected with LQTS as compared with asthma: child fear was measured with the R-CMAS and parental fear with the State-Trait Anxiety inventory. Children had comparable anxiety between groups but the mothers of LQTS patients had higher anxiety compared to those with severe asthma [60]. A study of children with ICDs (56% of the cohort affected by an ICC) was found to have a high prevalence of depression and anxiety symptoms as measured by the DISYPS-KJ [61] (see Table 1).

**Table 1:** Table 1 Quality of life and mental health studies in inherited cardiac conditions patients

Author/year/location	Aim	Study Population	Design	QoL/Mental Health measures	Findings
Czosek et al (2016) USA	Evaluate QoL in LQTs patients.	61 patients with LQTs	Quant cross sectional	Peds QL 4.0, PCQLI, (self and parent proxy), YSR (self), CBCL (parent)	Affected individuals significantly lower QoL, similar to Tet of Fallot, worse if internalizing symptoms present
Czosek e t al. (2015) USA	Evaluate QoL of children with arrythmic diseases, ICD effect on QoL.	288 patient pairs (107 with LQTs)	Quant cross sectional	PCQLI (self and parent proxy)	Lower QoL in LQTs patients with ICD Inserted
Spanaki et al. (2016) UK	Psychological wellbeing and QoL of HCM patients and impact of screening	108 Screening for FH of HCM, 23 G+P-, 21 affected by HCM	Quant cross sectional	Peds Ql 4.0, Peds QL cardiac module, strengths and difficulties questionnaire (self and parent proxy)	Affected children significantly lower QoL on generic and cardiac scores
Sleeper et al. (2016) USA	Measure QoL and functional status of patients with cardiomyopathy; determine if correlate with Sociodemographic, cardiac status and clinical outcomes	355 children with cardiomyopathy	Quant cohort study (from paediatric cardiomyopathy registry)	CHQ FSII(R) (parent proxy only)	All cardiomyopathy patients significantly lower physical functioning scores than normative data, lower QoL associated with adverse outcomes.
Friess et al. (2015) USA	Compare QoL of HCM patients with healthy controls, and patients with a family history of HCM	100 parents and 71 children	Quant cross-sectional	Peds QL 4.0 and Peds QL cardiac module (self and parent proxy)	Affected patients significantly lower QoL score compared to healthy controls and those at risk with family history, discrepancy between patient and parent scores
Bratt et al. (2013) Sweden	Compare QoL before and after diagnosis of HCM to healthy controls	13 affected, 41 controls	Quant longitudinal prospective case-control study	Lindstromm Questionnaire pre and post diagnosis. (self)	No difference in overall QoL following diagnosis, reduction in PA post diagnosis
Smets et al. (2008) Netherlands	Compare QoL of children with genetic conditions with normative data and compare parental proxy and self-reporting	35 patients genotype +ve for ICCs (LQT/familial hypercholesterolemia/HCM)	Quant cross sectional	Kidscreen 52 (self and parent proxy)	No difference between affected and normative data in QoL, parental rated emotional wellbeing significantly lower than self-report.
Last et al. (2018) UK	Evaluate CAQ in children, compare anxiety between those affected by ICCs, controls and those with a FH, examine if SCD in family increases anxiety	47 children with ICCs, 78 with a family history and 75 healthy controls	Quant cross section	CAQ-C, RCADS, CASI	Children with ICC significantly more cardiac anxiety than healthy controls and FH, FH children with SCD in family more anxious than affected children without a SCD
Patel et al. (2017) Canada	Study agreement and discrepancies between parental and child report of QoL and psychosocial health	50 parents and 50 affected children, 38 CHD, 5 arrhtymia, 4 cardiomyopathy, 3 inflammatory/infectious	Quant cross sectional	Peds Ql 4.0, BASC, multidimensional anxiety scale for children (self and parent proxy)	Moderate correlation for physical, school, social and overall psychosocial QoL, no associations for emotional QoL, internalizing problems, emotional skills, or anxiety.)
Giuffre (2008) Canada	Compare children with asthma to those with LQTs in terms of anxiety and medical fears	40 children with asthma and their mothers and 7 children with LQTs and their mothers	Quant cross sectional	FSSC- R,RCMAS (self), CBCL state-trait anxiety inventory (mothers)	Increased maternal anxiety compared to mothers with asthma, increased internalizing feelings in patients compared to asthma patients.
Eicken (2008) Germany	Measure the psychosocial impact of an ICD insertion	16 children with ICDs, 5 CHD, 9 ICC 2 myocarditis	Mixed methods	DISYPS-KJ, semi structured interviews	Depression and anxiety in half of the cohort including three patients with ICCs.

*BASC* behaviour assessment system for children, *CAQ-C* cardiac related anxiety questionnaire for children, *CASI* childhood anxiety sensitivity index, *CBCL* child behaviour checklist, *CHD* congenital heart disease, *CHQ* child health questionnaire, *DISYPS-KJ* depression and diagnostic system for psychiatric disorders in childhood and adolescence, *FSII* functional status tool, *ICC* inherited cardiac condition, *ICD* implantable cardioverter defibrillator; *LQTS* long QT syndrome, *HCM* hypertrophic cardiomyopathy, *PCQLI* paediatric cardiac quality of life inventory, *QoL* quality of Life, *RCADS* revised child anxiety and depression scale, *RCMAS* revised children’s manifest anxiety scale, *SCD* sudden cardiac death, *YSR* youth self report

#### Physical activity and fitness in ICC patients

Five studies were included that focussed solely on PA and cardiorespiratory fitness of children and young people affected by ICCs. Three were performed in LQTS and two in a mixed cohort of children affected by ICCs. Patients with ICCs were found to have reduced cardiorespiratory fitness as measured by both exercise stress testing [62] and cardiopulmonary exercise testing [63, 64] as well as reduced muscle mass [65] compared to healthy controls. Findings regarding PA were varied; some studies reported similar PA as measured by questionnaire [62] and accelerometery [65] between controls and affected individuals, whereas others found PA was less in the ICC group [63, 64]. An older study performed in 2013 found that, in a mixed cohort of children with ICCs, over 80% exceeded their advised physical restrictions when wearing an accelerometer [66] (see Table 2).

**Table 2:** Studies examining physical activity and fitness solely

Author/year/location	Aim	Study population	Design	PA/fitness measures	Findings
Gow (2013) Canada	Measure time ICC patients with activity restrictions spent in vigorous and very vigorous activity	13 patients with LQTs, 3 patients with CPVT	Cross sectional quantitative	Accelerometry measured time spent with METS > 7 and > 10	METS > 7 15/16 patients, (Med 113 min IQR 65–330), METS >/= 10 13/16 patients, (Med 53 IQR 9–115)
Boisson (2021) France	Measure fitness and adherence to exercise restrictions of ICC patients and adherence to WHO PA guidelines	32 patients with ICC	Cross sectional mixed methods	CPET, Ricci and Gagnon PA questionnaire, bespoke restricted activity questionnaire	Generally CPET good levels of aerobic fitness, 81% adhered to restrictions and 78% adhered to WHO PA guidelines, patients who exercised against restrictions > fitness than those who did not exceed restrictions
Souilla (2023) France	Compare physical fitness and PA of patients with LQTS compared with healthy controls	20 LQTS patients, 20 Controls	Prospective controlled cross sectional quantitative	CPET, accelerometry, muscles fitness (architecture by US and strength-hand grip and standing long broad jump)	LQTS patients had statistically lower Vo2 Max and VAT, lower grip strength and jumping distance, no significant difference in PA levels between LQTs and controls
Souilla (2025) France	Evaluate fitness and PA in ICC paediatric population and identify factors associated with Vo2 Max	100 patients with ICCs 107 controls	Prospective controlled cross-sectional study	CPET, accelerometry, Ricci and Gagnon PA questionnaire	Lower Vo2 Max and lower PA in affected patients, lower NYHA class, absence of an ICD, male sex, higher reported PA and pa motivation associated with higher Vo2 max in affected individuals
Chen et al (2022) Canada	Determine if PA levels are less in LQTs population compared with healthy controls	23 children with LQTs and 23 controls	Cross sectional	EST, PAQC/A	Significantly lower endurance time in LQT patients compared with controls, similar reported physical activity between groups

*CPVT* catecholaminergic polymorphic ventricular tachycardia, *CPET* cardiopulmonary exercise testing, *EST* exercise stress test, *ICC* inherited cardiac condition, *IQR* interquartile range, *LQTS* long QT syndrome, *Med* median, *METS* metabolic equivalent of task vat ventilatory anaerobic threshold, *PA* physical activity, *PAQC/A* physical activity questionnaire for children/adolescents, *VO2 Max* maximal oxygen consumption

#### Physical activity and its relationship to quality of life

##### Quantitative data

Multiple studies found that increased PA was associated with increased QoL Scores in young people affected by ICCs [57, 67, 68]. Correspondingly, PA restriction following diagnosis was often associated with lower QoL [69] or increased anxiety [70]. In some cases, a reduction in PA was not always associated with reduction in QoL; the authors suggested that this may reflect adaptation [55]. Positive parental outlook on child health correlated with increased activity time [68]. In adolescents with HCM increasing activity measured by increased kilometres walked or stairs climbed were both statistically significantly associated with higher physical QoL scores. However, it was not associated with higher psychological QoL scores [57]. Children with heart disease (38% of whom were affected by ICCs) reported higher QoL across all domains if they participated in sports [67].

Encouragingly, a pilot study performed in children with LQTS, and impaired cardiorespiratory fitness found that a supervised structured exercise programme could safely and successfully improve both cardiorespiratory fitness and strength as well as QoL scores [71]. Another pilot study has recently been conducted on an exercise programme in a carefully selected cohort of children with HCM. However, due to poor adherence and drop out, it was difficult to draw conclusions on improvement in cardiorespiratory fitness; there were no significant exercise related adverse events and a trend towards improved QoL and maximal working capacity in adherent participants [72] (see Table 3).

**Table 3:** Studies examining physical activity and quality of life

Author/year/location	Aim	Study population	Design	PA fitness measures and QoL measures	Findings
Gasior et al. (2023) Poland	Discover a causal diagram of sport participation in children and youth with heart disease	121 patients with heart disease (38% of cohort ICC)	Prospective cross-sectional study	PALMS, Peds QL 4.0, bespoke demographic/diagnosis questionnaire	Sports participants had higher Psychological QoL and physical health QoL Scores
Cunningham et al. (2020) Canada	Measure physical activity and identify risk factors associated with greater sedentary time	15 patients with CM	Prospective cross-sectional study	Accelerometry, HAES, CHQ	Reduced PA in cm patients compared to normative data; improved parental outlook on participants’ health correlated with less sedentary time. Participants taking part in sports greater PA time
Christian et al. (2020) Canada	Evaluate a cohort of patients with ICCS regarding physical and psychological well-being	35 children with ICCS	Prospective cross-sectional study	PEDs Ql 4.0, PCQLI, physical activity (MVPA)	Reduced PA compared to normative data, restrictions to PA is associated with lower QoL
Berg et al. (2018) USA	Evaluate the impact of sports restriction on asymptomatic adolescents with ICCs	12 children with ICCS	Prospective cross-sectional study, mixed methods	DIDs, Peds QL cardiac Module, SCARED	Correlation between adherence to activity restriction and increased anxiety noted but not statistically significant
Souilla et al. (2024) France	Assess feasibility, acceptability, safety and short-term benefits of cardiac rehabilitation in children and adolescents with LQTS and impaired cardiorespiratory fitness	Eight patients with LQTs (seven completed studies)	Feasibility pilot study, exercise intervention	Weight, height, BMI, HR, BP, echocardiography parameters, CPET, muscle fitness, accelerometry, Peds QL 4.0	Improved VAT, no change to VO2 max, improved muscular strength, improved QoL, no adverse outcomes
Wagner et al. (2024) Canada	Determine the relationship between Qol and PA in children with HCM	Fifty-six patients with HCM	Prospective multicentre cohort study	Accelerometry, Peds Ql 4.0	Reduced QoL compared to normative data, increased physical QoL domains associated with increased MVPA
Wanner et al. (2025) USA	Inform future trials on how to safely increase exercise capacity and physical activity in children with HCM	Eight patients with HCM (four completed Study)	Feasibility pilot study, exercise intervention	Peds QL 4.0. PCQLI, CPET	Poor adherence, improvement on QoL and maximal working capacity score in those who completed study

*BMI* body mass index, *BP* blood pressure, *CHQ* child health questionnaire, *CM* cardiomyopathy, *DIDs* dimensions of identity development scale, *HAES* habitual activity estimation scale, *HR* heart rate, *MVPA* moderate to vigorous physical activity, *PA* physical activity, *QoL* quality of life, *PCQLI* paediatric cardiac quality of life inventory, *SCARED* screen for anxiety related disorders, *VAT* ventilatory anaerobic threshold

##### Qualitative study themes

Seven studies were included that were either qualitative or mixed in methodology. The studies included cohorts of patients with one specific disease such as HCM, or patients with ICDs inserted, young athletes and patients with various type of ICCs who had been advised to restrict their activity. Several consistent themes emerged on reviewing the data. Children and young people’s experience of being diagnosed with an ICC varies, but it can be exceedingly difficult emotionally, with feelings of grief, unfairness, shock, and loss being common [73, 74]; younger age at diagnosis was protective [73]. Activity restriction was a universal theme [61, 70, 73–76]. This was often associated with frustration and a feeling of exclusion or being treated unfairly. The severity was particularly profound in young athletes [73, 74]. Other young people felt limited and concerned about aspects of their life, such as career options [70, 76].

Communication issues with healthcare providers and subsequent feelings of uncertainty were often described. Some young people and their parents felt that communication could have been improved both with regards to their diagnosis and procedures required for further investigation and management. Parents felt it was important that adolescents received an explanation of their diagnosis, even if simplified, from their doctors [74, 75].

Some studies suggested that fear of both cardiac events [70, 73] and ICD shocks [61, 75] was experienced by young people but was more overwhelming amongst parents, resulting in more severe activity restrictions than might be recommended by doctors for the children in some cases [76]. Children commented on their parents’ anxiety with regards to their condition and adherence to restrictions and medication [76].

Themes of resilience, coping and adjustment were also prevalent with affected individuals adapting to life with an ICC and in some cases even being thankful for the safety provided by being diagnosed and treated [73, 74]. Patients were reassured by encouragement from their doctors and felt that, by adhering to restrictions and taking their medicines/having an ICD inserted, they were protecting themselves, giving a sense of control [73, 76]. Participants described adjusting their physical activities, such as changing sports, changing the intensity with which they exercised or pursuing other interests [73, 74] (see Table 4).

**Table 4:** Common themes in qualitative studies on inherited cardiac conditions in children and young people

Study	Emotional response to diagnosis (e.g. grief, shock)	Activity restriction	Impact on future (career, lifestyle)	Fear of cardiac events or ICD shocks	Parental anxiety and influence	Communication and information needs	Resilience, coping, and adjustment
Eicken et al. (2006)		✓	✓	✓			
Berg et al. (2018)		✓	✓	✓		✓	
Bratt et al. (2012)	✓	✓	✓	✓			✓
Asif et al. (2015)	✓	✓	✓			✓	✓
Boisson et al. (2021)	✓	✓		✓	✓	✓	✓
Rahman et al. (2012)		✓		✓	✓	✓	✓
Meulenkamp et al. (2008)		✓	✓	✓	✓		✓

✓ = theme present in the study, *ICC* inherited cardiac condition, *ICD* implantable cardioverter defibrillator

## Discussion

This scoping review aimed to map the evidence regarding the impact of a diagnosis of an ICC on both PA and QoL. Many included studies found that those children with a diagnosis of an ICC had reduced QoL in comparison to healthy populations [50–54]. Symptoms associated with mental health disorders, such as anxiety and depression, were common in both quantitative and qualitative studies [32, 61]; the method of measurement varied widely between studies, reflecting researcher choice and practical considerations (cost/availability/translation of specific tools). The reported levels of PA varied but was lower in patients with ICCs, as was measured PA levels [57, 68, 77]; correspondingly, children with ICCs had lower cardiorespiratory fitness and muscular strength compared to controls [63, 65]. A strong link was found between PA and QoL across multiple studies, especially physical health reports [57, 67]. Content analysis of available literature included common themes of shock or grief at being diagnosed, activity restriction, desire for improved communication with healthcare providers and, lastly, resilience and coping [61, 70, 73]. These qualitative insights may help explain quantitative findings, where lower PA and QoL often appear linked to parental anxiety and strict activity limitation.

Previous reviews have charted and assessed the impact of both testing for and receiving a diagnosis of an ICC on QoL and mental health [78, 79]. To the authors’ knowledge, this is the first review to also map the evidence with regards to physical activity. This is timely as experts who care for children with ICCs begin to reassess the risk profile of PA and competitive sports, leading to more lenient practice [9, 80, 81]. Reduced QoL and poor mental health are commonly reported in patients with inherited cardiac conditions; however, those who engage in sports often demonstrate higher self-reported and parent-proxy QoL. The directionality of this association remains unclear and warrants further investigation. It is encouraging that improvement has been seen in two pilot studies of LQTS and HCM for both fitness and QoL [71, 72]; although small studies with highly selective inclusion criteria; they exemplify the potential of personalised medicine in this population. For this to be successful, a key group to reassure will be parents and guardians whose anxiety can outweigh that of the affected children [64]. In addition, clear, appropriate communication with young people needs to be prioritised as this has been highlighted as an issue that can affect long-term therapeutic relationships [75]. Clear and honest communication regarding the known and unknown risk with parents and young people are key pillars to shared decision making the approach advocated by ICC experts [82].

Studies tended to be conducted in children with more common diagnoses including LQTS and HCM; underrepresented conditions included CPVT and Brugada. This is unsurprising given they are rarer conditions [19]. Studies were predominantly cross-sectional in design with only a small number having longer term follow-up [73]. Many qualitative studies were retrospective in nature with participants being asked to recall past feelings and responses, which are prone to recall bias. Lastly, all the included studies were performed in high income countries, with predominantly white participants (ethnicity was reported in a minority of studies) (see Fig. 1) [83]. Therefore, research gaps to address in the future include rarer diseases, utilising prospective longitudinal design over a wider geographical area with more diverse ethnic groups. Some of these goals are ambitious as by their very nature much healthcare infrastructure, investment, and expertise are required to diagnose ICCs including but not limited to genetic laboratories and advanced radiological facilities (magnetic resonance imaging and echocardiography). These gaps will likely remain until affordable diagnostic solutions are more available and are made a priority for low- and middle-income countries.

This review benefits from both quantitative and qualitative data allowing a broad map of the existing evidence on this topic. For a rare group of conditions, there was a reasonable yield of articles. Limitations include the use of English only-publications. There is a possibility some studies were not included due to variations in terminology used. No formal critical appraisal or risk of bias was performed, as this was considered beyond the scope of this review. Additional limitations include the heterogeneity of QoL and PA tools, small and variable sample sizes, and the absence of a formal quality appraisal. These factors limit cross‑study comparability and the strength of our conclusions. Systematic reviews focused on (1) PA and cardiorespiratory fitness and (2) quality of life outcomes in children with inherited cardiac conditions would be valuable directions for future research.

## Conclusion

Children and young people with ICCs experience reduced QoL and mental health challenges which may be exacerbated by overly restrictive PA restrictions. Encouraging individualized, risk‑assessed physical activity may help improve QoL while maintaining safety. Adoption of standardized QoL and PA measures in future studies will enhance comparability and support evidence‑based recommendations for clinical care. Building on existing evidence would involve using objective physical activity data (e.g. wearable accelerometery, exercise stress testing, or cardiopulmonary exercise testing) alongside validated questionnaire tools. These tools, such as the PedsQL 4.0, PedsQL 3.0 Cardiac Module, and PCQLI, have been applied in multiple studies in this population and are likely to capture aspects of the themes identified in qualitative research. Continued efforts to improve the quality of research with consistent outcome measurement and family engagement should be a priority for researchers to help understand the scale of the issue and improve the patient experience.

## Supplementary information

Below are the links to the electronic supplementary materials.

Supplementary file 1 (DOCX 25.4 KB)

Supplementary file 2 (DOCX 90.2 KB)

Supplementary file 3 (PDF 651 KB)

## Data Availability

No datasets were generated or analysed during the current study.
