# Supplementary material for: “What impact does having a diagnosis of an inherited cardiac condition have on children and young people’s physical activity and quality of life?” A scoping review
Source: Eur J Pediatr. 2026 Jan 6;185(1):55. doi: 10.1007/s00431-025-06658-9 (PMC12775098; doi:10.1007/s00431-025-06658-9)
Supplement: Supplementary file 1 — (DOCX 25.4 KB) [file 431_2025_6658_MOESM1_ESM.docx]

Scoping Review Appendix 1 Searches

Embase <1974 to 2025 March 24>

1 physical activity/ 252634

2 exercise/ 381713

3 sport/ 61659

4 "quality of life"/ 715785

5 QOL.mp. 111545

6 genetic disorder/ 73467

7 inherited cardiac condition.mp. 93

8 long QT syndrome/ 13706

9 cardiomyopathy/ 77218

10 1 or 2 or 3 633889

11 4 or 5 729624

12 6 or 7 or 8 or 9 162226

13 10 and 11 and 12 378

14 child/ 2285004

15 young.mp. 1329628

16 adolescent/ 1917244

17 pediatrics/ 97605

18 paediatrics.mp. or pediatrics/ 112088

19 14 or 15 or 16 or 17 or 18 4252258

20 13 and 19 74

Ovid MEDLINE(R) ALL <1946 to March 24, 2025>

1 physical activity.mp. or Exercise/ 262919

2 Sports/ 36880

3 "Quality of Life"/ 302415

4 QoL.mp. 61781

5 Genetic Diseases, Inborn/ 14805

6 inherited cardiac conditions.mp. 107

7 Long QT Syndrome/ 9243

8 Cardiomyopathies/ 35235

9 Child/ 2012133

10 Adolescent/ 2315432

11 paediatrics.mp. 13656

12 Pediatrics/ 59593

13 1 or 2 292881

14 3 or 4 319607

15 5 or 6 or 7 or 8 59191

16 9 or 10 or 11 or 12 3321419

17 13 and 14 and 15 and 16 4

[**Accessibility Information and Tips**](javascript:openWideTip('https://support-ebsco-com.qub.idm.oclc.org/help/?int=ehost&lang=en&feature_id=access&TOC_ID=Always&SI=0&BU=0&GU=1&PS=0&ver=&dbs=ccm%27))

# **Print Search History**

| 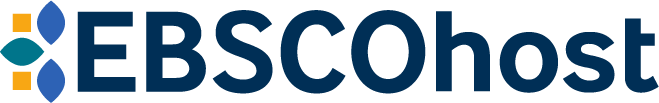 | Mon, March 24, 2025 12:35:28 pm |
| --- | --- |

| **#** | **Query** | **Limiters/Expanders** | **Last Run Via** | **Results** |
| --- | --- | --- | --- | --- |
| S1 | "physical activity OR ( exercise or physical fitness or physical activity ) AND ( quality of life or well being or well-being or health-related quality of life ) AND ( Genetic Diseases, Inborn OR Inherited Cardiac Condition OR Long Qt Syndrome OR Cardiomyopathy ) AND ( Child OR Young OR Adolescent OR Paediatrics OR Pediatrics )" | Limiters - Full Text; References Available; Abstract Available Expanders - Apply equivalent subjects Search modes - SmartText Searching | Interface - EBSCOhost Research Databases Search Screen - Advanced Search Database - CINAHL Complete | 825 |

Scopus (24^th^ March 25)

1Physical Activity OR Exercise OR Sports

AND

2 Quality of Life OR QoL

AND

3 Genetic Diseases, Inborn OR Inherited Cardiac Condition OR Long Qt Syndrome OR Cardiomyopathy

AND

4 Child OR Young OR Adolescent OR Paediatrics OR Pediatrics

Searched 1, 2, 3 & 4 (Results limited to English, from 1956 onwards)

Psych Info (24^th^ March 2025)

1Physical Activity OR Exercise OR Sports

AND

2 Quality of Life OR QoL

AND

3 Genetic Diseases, Inborn OR Inherited Cardiac Condition OR Long Qt Syndrome OR Cardiomyopathy

AND

4 Child OR Young OR Adolescent OR Paediatrics OR Pediatrics

Searched 1, 2, 3 & 4 (Results limited to English, from 1967 onwards)

Web of Science (24^th^ March 2025)

1Physical Activity OR Exercise OR Sports

AND

2 Quality of Life OR QoL

AND

3 Genetic Diseases, Inborn OR Inherited Cardiac Condition OR Long Qt Syndrome OR Cardiomyopathy

AND

4 Child OR Young OR Adolescent OR Paediatrics OR Pediatrics

Searched 1, 2, 3 & 4 (Results limited to English, from 1970 onwards)
