# Supplementary material for: “What impact does having a diagnosis of an inherited cardiac condition have on children and young people’s physical activity and quality of life?” A scoping review
Source: Eur J Pediatr. 2026 Jan 6;185(1):55. doi: 10.1007/s00431-025-06658-9 (PMC12775098; doi:10.1007/s00431-025-06658-9)
Supplement: Supplementary file 2 — (DOCX 90.2 KB) [file 431_2025_6658_MOESM2_ESM.docx]

Data Extraction Scoping Review (Supplemental Table 1)

| Authors | Berg et al. | Boisson et al. | Gąsior et al. | Bratt et al. | Bratt et al. |
| --- | --- | --- | --- | --- | --- |
| Year | 2018 | 2021 | 2023 | 2013 | 2012 |
| Design | Prospective cross-sectional | Observational cross-sectional | Observational prospective | Prospective Case Control | Descriptive qualitative Interview |
| Sample Size | 12 (of 30 eligible) | 32 (of 33 eligible) | 121 caucasien patients  56 (46%) sports participants  65 (54%) non-sports participants | 13 affected 41 controls | 13 asymptomatic children with HCM |
| Country | USA | France | Poland | Sweden | Sweden |
| Setting | Paediatric cardiology clinic, University of Utah | 2 Paediatric tertiary hospitals | Paediatric cardiology clinics, Warsaw | Inherited Cardiac Conditions Clinic, Gothenburg | Inherited Cardiac Conditions Clinic, Gothenburg |
| Methods | Mixed Methods | Mixed | Quantitative | Quantitative | Qualitative |
| Participant characteristics | 12–18 years (mean 14.7), 67% male  5 HCM, 1 DCM, 6 LQTS  Mean time since diagnosis 7.1 years | Mean 12.7 (±3.5), 65.6% male, 6 56.3% LQTS, Brugada, ARVC, HCM, DCM, CPVT  90% on beta blockers | 8–17 years 41% female, 59% male  Congenital Heart Disease, arrhythmia, cardiomyopathy, myocarditis | 5-18 years (median 11 years) 85% affected male,  New diagnosis of HCM asymptomatic | 8-18 (median 11) 85% male |
| Outcome Measures | PedsQL 4.0, (QoL)  SCARED, (Anxiety)  DIDS (Identity)  Bespoke Survey  Semi Structured Interview | CPET (VO2), Physical activity questionnaire, ESC guideline adherence questionnaire | PedsQL 4.0, PALMS-Y (motivation questionnaire), Causal inference Data driven diagram. | Lindström QoL Score | Not Applicable |
| Key Findings | Quantitative   1. Trends towards increased anxiety in adolescents adhering strictly to sport restrictions (R2 0.299) 2. No significant difference in QoL or identity domains.   Qualitative  1) restriction affects social/future career plans. | Quantitative   1. 81.3% adhered to 2020 ESC guidelines; the rest did not restrict their activities 2. 78.1% Adhered to WHO guideline 3. Good aerobic fitness in whole group 4. Peak HR< in adherent group (0.03)   Qualitative   1. Themes of frustration, adaptation to restrictions, resilience and positive impact of sports. | 1. Sport participation linked to better QoL and motivation enjoyment. 2. Causal links identified: age → sport → PE → enjoyment → QoL. | 1. Reduced physical activity compared to pre-screening in affected individuals 2. No significant difference in QoL | Themes  **Involuntary Change**  Grief, fear, gratitude  **Impact on daily Life**  Limitations, Tangibility, Adaptation, Social changes  **Reorientation**  Sense of control, Responsibility, Hope and faith in the future |
| Tools Validated (Y/N) | Yes | Yes (other than survey)  Thematic analysis referenced | Partially validated | Yes | N/A |
| Limitations | Small Sample size | Small Sample size | Heterogenous sample (congenital heart disease and inherited) Sample characteristics do not clear from description, Unsure of sport discipline | Small sample size | Same ethnic background of participants |

| Authors | Souilla et al. | Souilla et al. | Souilla et al. | Christian et al. | Wagner et al |
| --- | --- | --- | --- | --- | --- |
| Year | 2023 | 2024 | 2025 | 2020 | 2025 |
| Design | Prospective Controlled Study | Pilot study | Cross Sectional, Multi-Centre prospective control study | Cross Sectional Prospective Study | Multicentre prospective observational cohort study |
| Sample Size | 20 LQTs patient's vs 20 Healthy Controls | 8 children with LQTs (7 Completed study) | 100 affected children and 107 controls | 35 children affected by various ICCs | 56 children with HCM |
| Country | France | France | France | Canada | Canada |
| Setting | 2 Tertiary Paediatric Cardiology Department  CPET Labs  Physical Activity Centre  Strength Testing facilities | Paediatric Cardiac Rehabilitation centre | Seven tertiary care expert centres for inherited cardiac disease | Tertiary Paediatric Cardiology Department | Ten paediatric Cardiology Centres in Canada. |
| Methods | Quantitative | Quantitative | Quantitative | Quantitative | Quantitative |
| Participant characteristics | LQTS 12.7 +/- 3.7 years, Sex Ratio M: F 1.3, & Controls 11.9 +/- 2.4, Sex Ratio M: F 1.5 | 6–18-year-old with LQTS & impaired cardiorespiratory fitness  88% male | Affected by an ICC  12.7 +/- 3.1 years, 52% male, Controls 11.7+/- 3.3 50. %% male | Affected by an ICC  Age 12.3 +/- 3.2  57% Male | 15.5 Years (IQR13.8-16.8)  29% Female 71% Male  Primary HCM Diagnosis  30.4% ICD  12.5% Myomectomy |
| Outcome Measures | Vo2 Peak, Muscular Fitness, Activity levels (accelerometer measured) | Effect of 12-week exercise programme on  Weight, height, BMI, HR, BP, Echo parameters, VO2 peak, Ventilatory anaerobic threshold (VAT), Muscle fitness, Level of physical activity, Peds QL (parent and child reporting) | VO2 Max, Physical Activity (MVPA), Peds QL, Ricci & Gagnon PA questionnaire, motivational for health-orientated physical activity questionnaire  Social Demographic features | Peds QL  PCQLI  Physical Activity (MVPA)  Bespoke questionnaire about physical restriction impact | Peds QL  Physical Activity (MVPA) |
| Key Findings | VO2 Peak Less in LQTs participants compared with controls.  Reduced muscular fitness in LQTs participants compared with controls,  No significant difference in physical activity levels between LQTs and controls | Improved VAT (+24%),  Improved grip strength, Improved lower limb explosive strength, improved parent reported HRQOL no difference in muscle fitness/ PA outcomes, | Affected patients have significantly lower Vo2 max, engage in less physical activity, (even after adjusting for beta blockade) Have more sedentary time. | Affected patients engaged in less PA when compared to normative data.  Reduction in physical activity was associated with lower HRQoL  35 mins per day vs 55 mins per day  PEDs QL (79 Vs 84)  51% reduced their PA | Reduced HRQOL compared to normative data and (similar to children affected by other chronic conditions)  PEDS QL Physical health score associated with increased measured PA. |
| Tools Validated (Y/N) | Y | Y | Y | Y | Y |
| Limitations | Pilot Study  Beta Blockers and deconditioning | Small sample, No control group | Heterogenous sample | Small heterogenous sample  Compared to normative data | Modest Sample |

| Authors | Cunningham | Czosek | Czosek | Smets | Gow |
| --- | --- | --- | --- | --- | --- |
| Year | 2020 | 2016 | 2015 | 2008 | 20213 |
| Design | Prospective Cross Sectional | Prospective Cross Section  (Corollary Study) | Prospective Cross Sectional | Prospective Cross Sectional | Prospective Cross Sectional |
| Sample Size | 15 children with cardiomyopathy  14 with usable data | 61 patients with LQT | 118 patients with LQT | 35 patients genotype +ve for ICCs | 16 children with ICCs (13 LQT and 3 CPVT) |
| Country | Canada | USA | USA/UK | Holland | Canada |
| Setting | Paediatric Cardiology Clinic | 2x paediatric cardiology centres | 11 paediatric cardiology centres | 1 cardiogenetic clinic | Paediatric Cardiology Clinic |
| Methods | Quantitative | Quantitative | quantitative | Quantitative | Quantitative |
| Participant characteristics | Male 9 (64.3%)  Age 9.71 ± 4.34  Mixed Cardiomyopathy Cohort  50% recreational sports  NYHA Class 1/2 | Male 49%  Age 13.6+/- 3.0  LQT | Male 54%  Age 12.8 ± 3.0 years  LQT | 43% Male  Age 8-18  LQT/Familial Hypercholesterolemia/ HCM | Range 9-16  55% male  88% Activity restriction suggested by doctor |
| Outcome Measures | HRQOL  HAES  CHQ  PA (MVPA) | PEDS QL  PCQLI  YSR  CBCL | PCQLI | KIDSCREEN 52 | Accelerometery |
| Key Findings | Reduced PA in comparison to normative data amongst Cardiomyopathy patients (<MVPA).  Parents overestimate child's activity.  Improved parental outlook on Childs health correlated with less sedentary time. | Reduced generic and cardiac specific QoL scores when compared with normative data.  Similar scores to patients with Tetralogy of Fallot.  Symptoms on a beta blocker and presence of a Defibrillator associated with greater perceived disease impact | Patients with LQT with ICD lower QoL than those without | No Significant difference in QoL between affected and controls | Activity restrictions routinely exceeded with 13/16 81% of participants exceeding 10 mets during daily activities. |
| Tools Validated (Y/N) | Y | Y | Y | Y | Y |
| Limitations | Small Cohort  Heterogenous | Normative data |  | Heterogenous small sample | Modest sample Size |

| Authors | Spanaki et al. | Last et al | Sleeper et al | Friess et al. | Meulenkamp et al. |
| --- | --- | --- | --- | --- | --- |
| Year | 2016 | 2018 | 2016 | 2015 | 2008 |
| Design | Cross Sectional | Cross Sectional | Prospective Cohort Study | Cross Section | Cross Section Qualitative |
| Sample Size | 108 Screening for FH  23 G+P-  21 affected by HCM | 47 children with ICC  78 children with family history  75 controls | 355 children with cardiomyopathy | 100 parents of children with/screened for cardiomyopathy  71 children | 33 children |
| Country | UK | UK | US | US | Holland |
| Setting | Cardiogenetic Clinic | Cardiogenetic Clinic | 12 paediatric cardiology centres | Tertiary Paediatric Cardiology Centre | Cardiogenetics Clinic |
| Methods | Quantitative | Quantitative | Quantitative | Quantitative | Qualitative |
| Participant characteristics | Screening FH  67% male, Age 11.7+/- 2.96  G+p-  52% male, Age 11.8 +/- 3.53  Affected  76% male, Age 13.5 +/-2.8 | ICC  45% male  13.18 S. D 2.34  Family History  48% male  12.9 S. D 2.58  Controls  36% male  12.08 SD 2.39 | 149 DCM  50% Male  5.7(IQR 0.4,12.5)  129 HCM  71% Male  (IQR 3.8,14.3)  77 Assorted other cardiomyopathies  53% Male  9.2 (IQR 3.3,13.4) | 2 ARVC, 16 DCM, 4 RCM, 23 HCM, 22 LVNC 33 FH  55% Male  12 (S.D. 5) | 11 LQTS  6 HCM  16 Familial Hypercholesterolaemia |
| Outcome Measures | PEDSQL  Cardiac PEDs QL  Strengths and Difficulties | CAQ-C  CASI  RCADS | CHQ  FSII(R) | PEDSQL  PEDS QL Cardiac module | N/A |
| Key Findings | Children affected by Cardiomyopathy had significantly lower QoL Scores (both generic and cardiac specific) compared to children undergoing screening | Affected children are significantly more anxious about their heart compared to those with a FH & controls.  Children with a SCD in family significantly more anxious than those affected who had not (FH cohort not affected cohort) | All cardiomyopathy patients significantly lower physical functioning scores than normative data.  Lower physical CHQ summary scores associated with adverse outcomes. | Cardiomyopathy patients significantly lower QoL compared with normative data.  Patients with FH only similar QoL score compared to those affected by cardiomyopathy, contrasting with parental report which rated total QoL higher in FH than affected patients | Themes of Frustrations with being different, e.g. parents staying during swimming.  Increased breaks during exercise due to symptoms Some children refraining from sports all together due to parental concern.  Parents divided between lenient approach and very controlling with regards to physical activity. |
| Tools Validated (Y/N) | Yes | CASI/RCADS Y  CAQ-C (First time validated in this paper) | Yes | Yes | Y |
| Limitations | One clinic  Homogenous sample |  | Large cohort  Low event rate in some phenotypes | Heterogenous Cohort  Modest Sample Size | Small Sample  Families who agreed to be interviewed introduces bias |

| Authors | Asif et al. | Rahman et al. | Patel et al | Wanner et al | Giuffre et al. |
| --- | --- | --- | --- | --- | --- |
| Year | 2015 | 2011 | 2017 | 2025 | 2008 |
| Design | Cross Section Interview | Cross Section Interview | Cross Section | Pilot Study | Cross Section |
| Sample Size | 25 athletes | 6 patients and 6 parents | 50 patients and 50 parents | 8 patients with HCM recruited 4 completed study | 40 children with asthma and their mothers and seven children and their mothers with LQTs |
| Country | USA | Australia | Canada | USA | Canada |
| Setting | Telephone Interview  Recruited from various sources (Sports screening/ cardiology clinic) | Paediatric Cardiology Clinic | Paediatric Cardiology Clinic | Inherited Paediatric Cardiology Clinic | Paediatric Cardiology Clinic |
| Methods | Qualitative (Semi Structed Interviews) | Qualitative (Semi Structured Interviews) | Quantitative | Quantitative | Quantitative |
| Participant characteristics | 25 athletes  5 HCM 8 WPW 4 LQT  3 ASD 2 SVT 3 Other  Aged 14-35 at diagnosis | 1 CPVT, 1 LQTs, 3 Cardiomyopathy 1 Brugada Syndrome]  (15-17)  50% male | 38 CHD, 5 Arrythmia, 4 Cardiomyopathy  3 inflammatory/ infectious | 75% Male  Age range 8-18  NYHA I/II | 7 LQTS Patients  Mean age 13.71 Years (SD 2.14)  Asthma 11.10 Years (SD2.88) |
| Outcome Measures | NA | NA | PQLI, BASC, Multidimensional Anxiety Scale for Children | CPET  Peds QL  PCQLI | FSSC- R, RXMAS, CBCL State-Trait Anxiety Inventory |
| Key Findings | 4 stages of psychological impact and development   1. Immediate reactions and challenge to identity 2. Grief/coping 3. Adaptation 4. Acceptance   Risk factors higher level of competition, permanent disqualification, persistent reminders e.g. medicine, monitoring HR during activity | Difficulty with physical activity restrictions.  Feeling “not normal”  Poor communication of professionals.  Learning to cope with an ICD  Fear of being shocked  Placing limits on themselves | Parental and child agreement for  Physical, School, Social and overall psychosocial QoL.  No Significant associations for emotional QoL, internalising problems, personal adaptation or anxiety. | Very variable adherence to structured exercise programme varying from 1.25% to 100%.  No cardiac Related Adverse events  Trend towards improvement in QoL metrics and Cardiorespiratory fitness however not analysed due to low numbers. | Increased Maternal anxiety compared to mothers with asthma.  Increased internalising feelings in LQTs patients compared with asthma. |
| Tools Validated (Y/N) | Y | N/A | Y | Y | Y |
| Limitations | Small heterogenous sample | Very Small sample | Heterogenous sample | Small study, High dropout range | Very Small LQTs sample compared with moderate Asthma |

| Authors | Eicken et al | Chen et al |  |  |  |
| --- | --- | --- | --- | --- | --- |
| Year | 2006 | 2022 |  |  |  |
| Design | Mixed Methods Cross Sectional | Cross Sectional |  |  |  |
| Sample Size | 16 children with ICD | 23 children with LQTS and 23 PAQ Controls |  |  |  |
| Country | Germany | Canada |  |  |  |
| Setting | Paediatric Cardiology Department | Paediatric Cardiology Department |  |  |  |
| Methods | Mixed | Quantitative |  |  |  |
| Participant characteristics | 16 children with ICDS  5 CHD, 9 ICC 2 Myocarditis | 23 LQT Children Mean Age 14.7+/- 0.5  43% male  23 controls  14.5 +/-0.4 |  |  |  |
| Outcome Measures | DISYPS-KJ  Semi Structured Interviews | PAQ-C  Exercise Stress Testing |  |  |  |
| Key Findings | Depression and Anxiety in half of the cohort including three patients with ICCs.  Themes from interviews  Reduction in physical activity levels  Fear of further shocks | Similar levels of Physical Activity reported by LQT patients as controls.  Lower peak HR of LQTs patients (91% on beta blockers)  Significantly lower Exercise time in LQTs group.  Reported PA Time similar between both cohorts, neither LQTs nor Cohorts meeting PA activity guidelines. |  |  |  |
| Tools Validated (Y/N) | Y | Y |  |  |  |
| Limitations | Small heterogenous cohort | Small Cohort, two control groups,  Beta Blockade potential confounder |  |  |  |
